# Cystic Dilation of Extrahepatic Bile Ducts in Adulthood: Diagnosis, Surgical Treatment and Long. Term Results

**DOI:** 10.1155/1998/90178

**Published:** 1998

**Authors:** G. Belli, G. Rotondano, A. D'Agostino, A. Iannelli, I. Marano, M. L. Santangelo

**Affiliations:** 1 Department of General Surgery & Organ Transplantation University of Naples “Federico II” School of Medicine Naples Italy; 2 Department of Radiology University of Naples “Federico II” School of Medicine Naples Italy

## Abstract

To evaluate the long-term results of surgery for choledohal cyst in adulthood, a series of 13 patients over the age of 16 operated on for choledochal cyst during a period of six years and followed-up for a minimum of 3 years was analyzed. Patients with type I and IVa cysts underwent extrahepatic cyst resection and Roux-en-Y hepatico-jejunostomy. Choledochoceles (type III) were managed endoscopically. No operative mortality or morbidity occurred. Type I and III cysts showed almost ideal follow-up with no sign of stricture on HIDA scan. One type IVa cyst patients developed recurrent cholangitis due to anastomotic stricture, managed percutaneously. Whenever possible, complete cyst resection and Roux-en-Y reconstruction is the treatment of choice for all extrahepatic biliary cysts. Intra- and extrahepatic dilatations are adequately treated by extrahepatic resection and careful endoscopic or radiologic surveillance. Small choledochoceles can be safely managed by endoscopic sphincterotomy.


*HPB Surgery*, 1998, Vol. 10, pp. 379–385
Reprints available directly from the publisher
Photocopying permitted by license only

© 1998 OPA (Overseas Publishers Association)
Amsterdam B.V. Published under license
under the Harwood Academic Publishers imprint,
part of The Gordon and Breach Publishing Group.
Printed in India.

### Cystic Dilation of Extrahepatic Bile Ducts in Adulthood: Diagnosis, Surgical Treatment and Long Term Results

G. BELLI[a], G. ROTONDANO[a], A. D'AGOSTINO[a], A. IANNELLI[a], I. MARANO[b] and M. L. SANTANGELO[a]

[a]*Department of General Surgery & Organ Transplantation;* [b]*Department of Radiology, University of Naples "Federico II", School of Medicine, Naples, Italy*

*(Received 30 July 1996; In final form 2 January 1997)*

**To evaluate the long-term results of surgery for choledohal cyst in adulthood, a series of 13 patients over the age of 16 operated on for choledochal cyst during a period of six years and followed-up for a minimum of 3 years was analyzed. Patients with type I and IVa cysts underwent extrahepatic cyst resection and Roux-en-Y hepatico-jejunostomy. Choledochoceles (type III) were managed endoscopically. No operative mortality or morbidity occurred. Type I and III cysts showed almost ideal follow-up with no sign of stricture on HIDA scan. One type IVa cyst patients developed recurrent cholangitis due to anastomotic stricture, managed percutaneously. Whenever possible, complete cyst resection and Roux-en-Y reconstruction is the treatment of choice for all extrahepatic biliary cysts. Intra- and extrahepatic dilatations are adequately treated by extrahepatic resection and careful endoscopic or radiologic surveillance. Small choledochocelbs can be safely managed by endoscopic sphincterotomy.**

*Keywords:* Choledochal cysts, cholangiography, scintigraphy, surgery, follow-up

### INTRODUCTION

Cystic dilatation of the extrahepatic bile ducts are uncommon conditions which are being diagnosed more frequently with the improvement in biliary imaging techniques. While the underlying cause is probably congenital, the cysts might not present until adulthood. According to Todani classification [1], type I cysts are solitary fusiform extrahepatic dilatations, type III are intraduodenal diverticula (choledochoceles) and type IVa are fusiform extra- and intrahepatic cystic dilatations. Symptoms are related either to the size of the cyst or to the varying degrees of biliary obstruction with or without concurrent cholangitis. Although treatment of type I cysts is accepted by the surgical community, the management of type III and IV cysts remains controversial and will ary depending on the pre- and intraoperative

Correspondence to: Giulio Belli, M. D., Via Cimarosa 2/a, 80127 Naples, Italy. #Phone and fax: 39-81-7462550.

findings. We report 13 extrahepatic biliary cysts treated in adulthood and followed-up for a minimum of 3 years, discussing the relative clinico-diagnostic implications and reviewing the treatment options.

### PATIENTS AND METHODS

From January 1987 through December 1992, the records of all patients over the age of 16 affected with cystic dilatation of the extrahepatic biliary system were retrospectively analyzed. There were 13 such patients (9 females, 4 males) with a mean age of 30.8 years (range 17 – 55). During the study period three additional patients referred to our department were excluded because of younger age (under 16). The diagnosis was established by means of one or more of the following: ultrasonography (US), endoscopic retrograde cholangio-pancreatography (ERCP), percutaneous transhepatic cholangiography (PTC), computed tomography (CT) and magnetic resonance imaging (MRI). This last was employed only after 1990. The type of dilatation was classified according to Todani [1]. Follow-up programme included liver function tests and US at 6 and 12 months and yearly afterwards. All bilio-enteric anastomoses were evaluated with a $^{99m}$Tc-HIDA scintigraphy at 6 and 18 months. The results of surgery were considered good if patients were symptoms-free or had occasional (less than 2/year) mild episodes of cholangitis requiring only conservative therapy.

### RESULTS

There were 8 type I (62%), 2 type III (15%) and 3 type IVa (23%) choledochal cysts. The most common symptoms were abdominal pain (78%), jaundice (57%) and fever (28%). No patient had a palpable mass. Six patients (3 type I, 1 type III and 2 type IVa) had coexistent biliary stones (1 intrahepatic, 2 gallbladder stones and 3 biliary sludge within the cyst); 3 patients had received prior biliary tract surgery elsewhere (1 cholecystectomy and 2 cysto-duodenostomy) (Tab. I).

An abnormally long (>15 mm) common pancreaticobiliary duct junction (PBDJ) could be identified at ERCP only in 4 cases (31%). Both CT scan and MRI were valuable diagnostic tools. In particular, the cyst type as identified on MRI, always correlated with direct cholangiography and intraoperative findings; furthermore, when MRI ruled out intrahepatic involvement, this was always subsequently confirmed at operation.

Liver function tests in these patients showed increased alkaline phosphatase levels (mean 820 U/L with upper limits of 250 U/L) and GPT levels (mean 120 U/L with upper limits of 40 U/L). All patients with type I disease underwent total cyst excision with Roux-en-Y hepatico-jejunostomy, including reoperation in two patients who had had cysto-duodenostomy at age 3 and 6 respectively, complaining of recurrent cholangitis. There were no early complications and no patient has developed anastomotic stricture or cholangitis on a mean follow up of 77.1 months (range 38 – 122) as confirmed by normal post-operative HIDA scan. Postoperatively, only slight elevation of alkaline phosphatase levels persisted (two times normal values) and US confirmed no intrahepatic dilatation.

Clinical presentation in the 2 patients with choledochocele (type III) was pancreatitis and cholangitis, respectively. They were treated by endoscopic sphincterotomy and remain symptoms-free at 41 and 55 months, respectively. All three patients with type IVa disease underwent extrahepatic cyst excision at the level of the bifurcation of the left and right hepatic ducts with a Roux-en-Y hepatico-jejunosotomy. Fourteen months after operation, one of them had some evidence of recurrent cholangitis due to intrahepatic lithiasis over an anastomotic stricture identified on HIDA scan (delayed flow >60 minutes). She was therefore treated with percutaneous balloon dilatation, stone extraction and stenting with good results. She is in good health 46 months after primary operation, as are the other two patients at 58 and 103 months of follow-up respectively (Tab. I).

### DISCUSSION

Choledochal cysts are recognized as disease of childhood, however in recent years this condition has been reported in increasing numbers of adult patients [2, 3]. An abnormally long common PBDJ has been advocated as a possible etiological mechanism [4]. The low incidence of such abnormality in the present series (only 31%) is likely to be an underestimate as the Wirsung was not filled in 6 patients, precluding the possibility to assess further anomalies of the PBDJ.

Children and adults differ in clinical presentation, with adults commonly having acute

TABLE I Clinical features and treatment of choledochal cysts in 13 adult patients

| N | Sex | Age | Cyst type | Symptoms | Prior Surgery | Diagnosis | Treatment | Follow-up* |
|---|---|---|---|---|---|---|---|---|
| 1 | M | 20 | I | pain, ↑ GPT | no | US, ERCP, MRI | HJS | 70 |
| 2 | F | 23 | IVa | cholangitis | no | US, PTC, MRI | HJS** | 46 |
| 3 | M | 55 | I | pain, leukocytosis | no | At laparotomy | HJS | 77 |
| 4 | F | 26 | I | jaundice | no | US, ERCP, MRI | HJS | 49 |
| 5 | F | 29 | I | pain, ↑ amylase | no | US, ERCP, MRI | HJS | 38 |
| 6 | F | 18 | I | cholangitis | cystoduodenostomy | US, ERCP, | HJS | 81 |
| 7 | F | 41 | III | cholangitis | no | US, ERCP | ES | 55 |
| 8 | M | 39 | I | pain, ↑ GPT | cholecystectomy | ERCP, CT | HJS | 115 |
| 9 | F | 31 | III | jaundice, pain | no | US, ERCP | ES | 41 |
| 10 | M | 32 | IVa | pain, ↑ GPT | no | US, ERCP, CT | HJS | 103 |
| 11 | F | 21 | I | cholangitis | cystoduodenostomy | US, ERCP, CT | HJS | 122 |
| 12 | F | 37 | I | jaundice | no | US, ERCP | HJS | 94 |
| 13 | F | 32 | IVa | jaundice, pain | no | US, PTC, MRI | HJS | 58 |

*in months.
**recurrent cholangitis due to anastomotic stricture 14 mo. after surgery; percutaneous management with good results.
HJS: hepatico-jejunostomy; ES: endoscopic sphinterotomy.

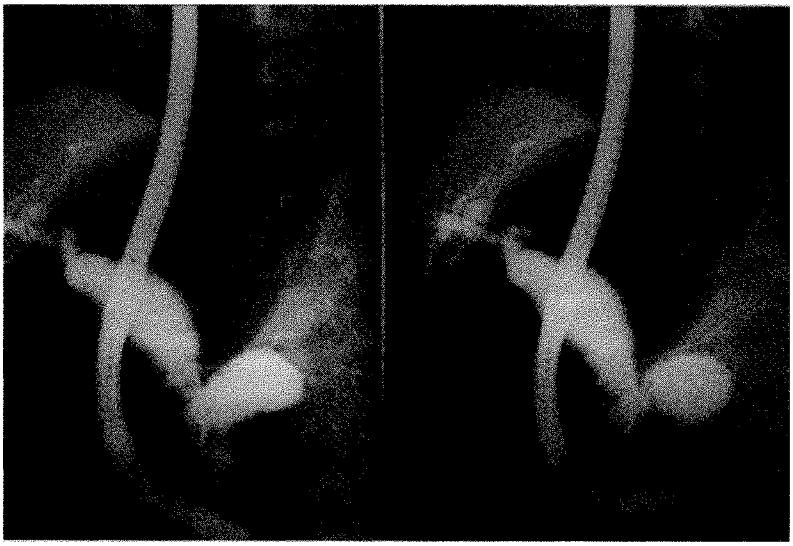

FIGURE 1 ERCP showing diffuse cystic enlargement of the common bile duct and intrahepatic ducts (type IVa). Pancreatography reveals a normal Wirsung.

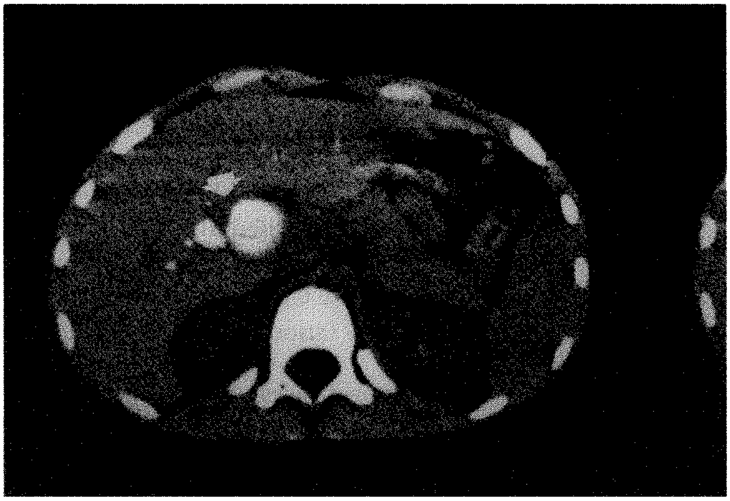

FIGURE 2 Cholangio-CT scan with good opacification of the biliary tree at the liver hilum and homogeneous cystic dilatation of the common bile duct (arrow).

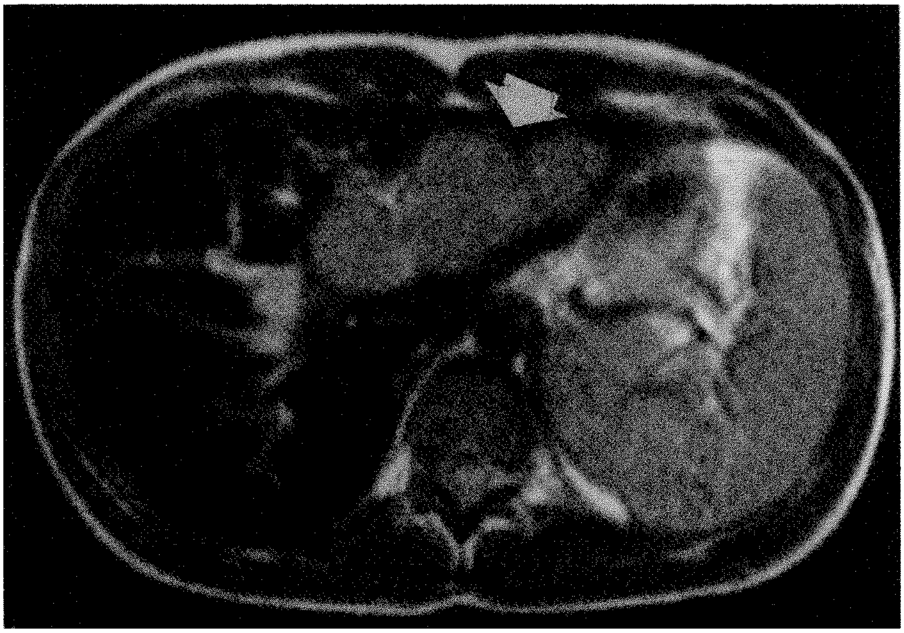

FIGURE 3 Abdominal MRI demonstrating marked dilatation of left biliary system up to the bifurcation (arrow). There are no signs of biliary stasis within the right liver.

bilio-plancreatic symptoms [5,6]. In our series cholangitis and epigastric pain were the predominant clinical features. Laboratory findings, although nonspecific, were useful in that they prompted deeper investigations in 5 cases. Approximately 20–60% of patients present in adulthood [7]; this may be partly caused by the late onset of symptoms and partly by the limited imaging methods in the past. US and CT scan are diagnostic; MRI may more accurately define the extent of intrahepatic biliary dilatation. Although only partially employed in our series, MRI showed a 100% correlation with cholangiographic features and with intraoperative findings. Direct cholangiography with both PTC or ERCP, is still essential to differentiate the type of biliary cyst and plan the extent of operative resection [4, 8, 9]. Nonetheless, revolutionary diagnostic contributions are awaited in the near future from the new 3D MRI-cholangiography, which is under evaluation at our institution. Isotopes should have little if any role, being most valid only in assessing anastomotic patency, as reported by our group [10].

Isolated fusiform dilatation of the extrahepatic biliary tree (type I) usually account for 50–80% of all cases [4, 9, 11–16]. Type IV are the second most commonly identifiable cyst type (10–25%) [14]. Type II and type III cysts are extremely rare.

The indication for surgery usually result from imminent complications or from the risk of malignant transformation (about 20 times more than the general population and increasing with age [17]). Bile duct cancer develops earlier when patients have previously undergone various internal drainage procedures, such as cyst-enterostomy and their prognosis is dismal [5, 13, 17]. Complete excision of the cyst can be accomplished with the same mortality and less morbidity than drainage procedures [2–7, 12–16]. Total surgical excision and biliary reconstruction with Roux-en-Y hepaticojejunostomy is now widely accepted as the standard of care in adults, choledocho-duodenostomy still being a valuable option in childhood. All type I cysts in our series underwent total excision followed by hepaticojejunostomy and showed almost ideal long-term follow-up results. Choledochoceles do not appear to have malignant potential nor to be full-thickness defects of the common bile duct. Thus adequate drainage can be generally achieved by endoscopic sphincterotomy with satisfactory results [18], surgical treatment is rarely indicated.

Treatment for type IVa cysts is still controversial. These cysts by definition have an intrahepatic cystic area and, therefore, complete cyst excision would require hepatic resection. Excision of the extrahepatic portion of the cyst with Roux-en-Y reconstruction provides adequate drainage and in many cases the intrahepatic dilatation may regress [19]. Still, patients remain at risk of recurrent cholangitis and later development of carcinoma, though malignancy in these patients is neither always intracystic nor prevented completely by cyst excision. Patients with type IVa cyst in our series showed no biliary cancer at 46, 58 and 103 months, respectively: our results and those of others [6, 12, 13, 20, 21]. suggest that extrahepatic cyst excision and careful surveillance may currently be the choice of treatment for this type of cysts. The chance of performing an "endoscopy-friendly" resection with a modified Hutson access loop should not be overlooked in this setting [22].

### References

[1] Todani, T., Watanabe, Y., Narusue, M., Tabuchi, K. and Okajima, K. (1977). Congenital bile duct cysts. Classification, operative procedures, and review of thirty-seven cases including cancer arising from choledochal cyst. *American Journal of Surgery*, **134**, 263–69

[2] Deziel, D. J., Rossi, R. L. and Munson, J. L. (1986). Management of bile duct cysts in adults. *Archives of Surgery*, **121**, 410–5.

[3] O' Neill, J. A., Templeton, J. M., Schnaufer, L., Bishop, H. C., Ziegler, M. M. and Ross III, A. J. (1987). Recent experience with choledochal cyst. *Annals of Surgery*, **205**, 533–40.

[4] Rattner, D. W., Schapiro, R. H. and Warshaw, A. L. (1983). Abnormalities of the pancreatic and biliary ducts in adult patients with choledochal cysts. *Archives of Surgery*, **118**, 1068–73.

[5] Nagorney, D. M., McIlrath, D. C. and Adson, M. A. (1984). Choledochal cysts in adults: clinical management. *Surgery*, **96**, 656–63.

[6] Scudamore, C. H., Hemming, A. W., Teare, J. P., Fache, J. S., Erb, S. R. and Watkinson, A. E. (1994). Surgical management of choledochal cysts. *American Journal of Surgery*, **167**, 497–500.

[7] Robertson, J. F. and Raine, P. A. (1988). Choledochal cyst: a 33 year review. *British Journal of Surgery*, **75**, 799–801.

[8] Savader, S. J., Benenati, J. F. and Venbrux, A. C. (1991). Choledochal cysts: classification and cholangiografic appearance. *American Journal of Roentgeno-logy*, **156**, 327–31.

[9] Wiedmeyer, D. A., Stewart, Et., Dodds, W. J., Geenen, J. E., Vennes, J. A. and Taylor, A. J. (1989). Choledochal cyst: findings on cholangiopancreatography with emphasis on ectasia of the common channel. *American Journal of Roentgenology*, **153**, 969–72.
[10] Belli, G., Romano, G., Monaco, A. and Santangelo, M. L. (1988). HIDA scan in the follow-up of biliary-enteric anastomoses. *HPB Surgery*, **1**, 29–34.
[11] Yamaguchi, M. (1980). Congenital choledochal cyst. Analysis of 1433 patients in the Japanese literature. *American Journal of Surgery*, **140**, 653–57.
[12] Benhidjeb, T., Munster, B., Ridwelski, K., Rudolph, B., Mau, H. and Lippert, H . (1994). Cystic dilatation of the common bile duct: surgical treatment and long-term results. *British Journal of Surgery*, **81**, 433–36.
[13] Chijiiwa, K. and Koga, A . (1993). Surgical management and long-term follow-up of patients with choledochal cysts. *American Journal of Surgery*, **165**, 239–42.
[14] Katyal, D. and Lees, G. M. (1992). Choledochal cysts: a retrospective review of 28 patients and a review of the literature. *Canadian Journal of Surgery*, **35**, 584–88.
[15] Lopez, R. R., Pinson, C. W. and Campbell, J. R. (1991). Variation in management based on type of choledochal cyst. *American Journal of Surgery*, **161**, 612–15.
[16] Nagata, E., Sakai, K., Kinoshita, H. and Hirohashi, K. (1986). Choledochal cyst: complications of anomalous connection between the choledochus and pancreatic duct and carcinoma of the biliary tracat. *World Journal of Surgery*, **10**, 102–10.
[17] Voyles, C. R., Smadja, C., Shands, W. C. and Blumgart, L. H. (1983). Carcinoma in the choledochal cysts. Age-related incidence. *Archives of Surgery*, **118**, 986–88.
[18] Martin, R. F., Biber, B. P., Bosco, J. J. and Howell, D. A. (1992). Symptomatic choledochoceles in adults. Endoscopic retrograde cholangio-pancreatography recognition and management. *Archives of Surgery*, **127**, 536–39.
[19] Thambi Dorai, C. R., Visanathan, R. and McAll, G. L. (1991). Type IVa choledochal cysts: surgical management and review of the literature. *Australian New Zealand Journal of Surgery*, **61**, 505–10.
[20] Schmid, C., Meyer, H. J., Ringe, B., Scheumann, G. F. and Pichlmayr, R. (1993). Cystic enlargement of extrahepatic bile ducts. *Surgery*, **114**, 65–70.
[21] Lipsett, P. A., Pitt, H. A., Colombani, P. M., Boitnott, J. K. and Cameron, J. L. (1994). Choledochal cyst disease. A changing pattern of presentation. *Annals of Surgery*, **220**, 644–52.
[22] Henne-Bruns, D., Kremer, B. and Thonke, F. *et al.* (1993). "Endoscopy-frdiendly" resection technique of choledochal cyst. *Endoscopy*, **25**, 176–178.

### INVITED COMMENTARY ON

Belli G, Rotondano G, D'Agostino A, Iannelli A, Marano I, Santangelo ML. Cystic dilatation of extrahepatic bile ducts in adulthood: Diagnosis, surgical treatment and long-term results.

M. D. Shahrudin and R. C. N. Williamson
Department of Surgery
Royal Postgraduate Medical School
Hammersmith Hospital
London W12 ONN
United Kingdom

### COMMENTARY

Although bile duct cysts are typically a paediatric surgical problem, in about 20% of patients the diagnosis is delayed until adulthood. The clinical presentation and therapeutic strategies differ at these two stages of life. Compared with children, adults have an increased rate of associated hepatopancreatobiliary pathology [1–3, 4] and they often present with complications of previous cyst-relataed procedures [3–7]. Despite the heterogeneity of the disease and the absence of clinical trials, a consensus of management for choledochal cysts has emerged in the last decade.

Dr Belli and colleagues report their experience with the management of congenital biliary dilatation diagnosed in adulthood, highlighting the value of endoscopy in treating choledochoceles. Several points need to be stressed about this article. We strongly believe that direct cholangiography is the best technique for accurate definition of the type of choledochal cyst [4]. Indeed, cyst classification is based upon cholangiographic features [8, 9]. Direct cholangiography, which is a prerequisite to operation, has the advantage of defining the configuration and extent of the cyst, the presence of stones within the gallbladder, bile duct, cyst or pancreatic duct and any ductal strictures or filling defects that might suggest malignant change. Complete visualisation of the entire pancreatobiliary tree is important in patients with choledochal cysts, because failure to recognise segmental areas of dilatation within the liver parenchyma or associated pancreatic duct anomalies may lead to sepsis, subsequent cholangitis, pain, pancreatitis and eventual re-operataion.

PTC and ERCP have their own advantages and disadvantages in the diagnosis of choledochal cysts. Adults without previous cyst-enterostomy are best evaluated by ERCP because it allows better visualisation of an abnormal pancreaticobiliary ductal junction (PBDJ). PTC is particularly advantageous in patients with previous Roux-en-Y cyst-enterostomy and in patients with type IV (intrahepatic) cysts, but it may fail to define clearly the pancreaticobiliary junction. An anomalous PBDJ could have been missed in the 2 patients who had PTC in the present series as well as in the 55-year-old man diagnosed at laparotomy, and this could explain the low incidence (31%) of this anomaly.

The rapiditdy and accuracy of ultrasound, combined with its ability to image adjacent viscera, support its use as the initial investigative procedure. CT scan combined with intravenous cholangiography can supplement this information. With time, comparative studies may show whether magnetic resonance cholangiopancreatography (MRCP) will replace direct cholangiography as the preferred diagnostic modality.

The usual treatment of type III bile duct cysts (choledochocele) in adults is either transduodenal excision or transduodenal sphincteroplasty [10], the choice being governed by the size of the choledochocele. Size is not mentioned in the present report.

The age-related risk of malignancy and the frequency of late anastomotic strictures in patients with type IV lesions warrant long-term postoperative follow-up. We favour an access loop as this could allow direct endoscopic inspection and biopsy of any suspicious lesions plus treatment of anastomotic strictures.

Shahrudin Mohd Dun
and Robin C. N. Williamson
Department of Surgery
Royal Postgraduate Medical School
Hammersmith Hospital
Du Cane Road
London W12 ONN
United Kingdom

### *References*

[1] Rattner, D. W., Schapiro, R. H. and Warshaw, A. L. (1983). Abnormalities of the pancreatic and biliary ducts in adult patients with choledochal cysts. *Archives of Surgery* **118**, 1068–1073.

[2] Nagomey, D. M., Mcllrath, D. C. and Adson, M. A. (1984). Choledochal cysts in adults: *clinical management. Surgery* **96**, 656–663.

[3] Schmid, C., Meyer, H. J., Ringe, B., Scheumann, G. F. and Pichlmayr, R. (1993). Cystic enlargement of extrahepadtic bile ducts. *Surgery* **114**, 65–70.

[4] Hopkins, N.F.G. Benjamin, I. S., Thompson, M. H. and Williamson, R.C.N. (1990). Complications of choledochal cysts in adulthood. *Annals of the Royal College of Surgeons of England* **72**, 229–235.

[5] Chijiiwa, K. and Koga, A. (1993). Surgical management and long-term follow-up of patients with choledochal cysts. *American Journal of Surgery* **165**, 239–242.

[6] Trout, H. H. and Longmire, W. P. (1971). Long-term follow-up study of patients with congenital cystic dilatation of the common bile duct. *American Journal of Surgery* **121**, 68–86.

[7] Todani, T., Watanabe, Y., Fujii, T., Toki, A., Uemura, S. and Koike, Y. (1984). Congenital choledochal cyst with intrahepatic involvement. *Archives of Surgery* **119**, 1038–1043.

[8] Matsumoto, Y., Uchida, K., Nakase, A. and Honjo, I. (1977). Clinico-pathologic classification of congenital cystic dilatation of the common bile duct. *American Journal of Surgery* **134**, 569–574.

[9] Todani, T., Watanabe, Y., Narusue, M., Tabuchi, K. and Okajima, K. (1977). Congenital bile duct cysts. Classifications, operative procedures, and review of thirty-seven cases including cancer arising from choledochal cyst. *American Journal of Surgery* **134**, 263–269.

[10] Okada, A., Nakamura, T., Higaki, J., Okumura, K., Kamata, S. and Oguchi, Y. (1990). Congenital dilatation of the bile duct in 100 instances and its relationship in anomalous junction. *Surgery, Gynaecology, and Obstetrics* **171**, 291–298.

